# Racial and Ethnic Disparities in Child Abuse Identification and Inpatient Treatment

**DOI:** 10.1001/jamanetworkopen.2024.51588

**Published:** 2024-12-18

**Authors:** Fereshteh Salimi-Jazi, Norah E. Liang, Zhuoyi Huang, Lakshika Tennakoon, Talha Rafeeqi, Amber Trickey, Stephanie D. Chao

**Affiliations:** 1 Division of Pediatric Surgery, Department of Surgery, Stanford University School of Medicine, Stanford, California; 2 Division of General Surgery, Department of Surgery, Stanford University School of Medicine, Stanford, California; 3 Stanford-Surgery, Policy, Improvement Research, and Education Center, Department of Surgery, Stanford University School of Medicine, Stanford, California

## Abstract

**Importance:**

Child abuse is a leading cause of morbidity in early childhood. Accurate detection remains challenging.

**Objective:**

To describe racial and ethnic disproportionalities in suspicion for child abuse (SCA) in pediatric patients admitted after traumatic injury.

**Design, Setting, and Participants:**

This retrospective, multicenter cross-sectional study performed between 2022 and 2024 examined a representative national sample of children and adolescents (aged <18 years) admitted for pediatric trauma indications from 2006 to 2019. Data were from the Kids’ Inpatient Database (KID), the largest publicly available all-payer pediatric inpatient care database. Patient demographics, injury severity, and hospitalization characteristics were classified by race and ethnicity and compared using univariate and multivariate regression. Statistical analysis was performed between March 2022 and October 2024.

**Exposure:**

The SCA subgroup was identified using *International Classification of Diseases, Ninth Revision, Clinical Modification *(*ICD-9-CM*) and *International Statistical Classification of Diseases, Tenth Revision, Clinical Modification *(*ICD-10-CM*) codes corresponding to child and adolescent maltreatment.

**Main Outcome and Measure:**

The primary outcome was the racial and ethnic composition of pediatric patients admitted after traumatic injury with SCA vs those without SCA.

**Results:**

Among the weighted total of 634 309 pediatric patients with complete data included in the study, 13 579 patients had injuries attributable to SCA (SCA subgroup; mean [SD] age, 1.70 [0.04] years; 7650 male [56.3%]; 2868 Black [21.1%], 2293, Hispanic [16.9%], and 5675 White [41.8%]) and 620 730 patients did not (non-SCA subgroup; mean [SD] age, 9.70 [0.01] years; 395 158 male [63.7%]; 86 376 Black [13.9%], 108 406 Hispanic [17.5%], and 298 748 White [48.1%]). The racial and ethnic distribution of the non-SCA subgroup was similar to that of the 2010 US Census. In the SCA subgroup, Black patients (odds ratio [OR], 1.75; 95% CI, 1.65-1.85; *P* < .001) and Hispanic patients were overrepresented (OR, 1.11; 95% CI, 1.05-1.18; *P* < .001) and White patients were underrepresented compared with the Census. After controlling for socioeconomic factors and hospital characteristics among 504 365 (weighted) observations in White, Black, and Hispanic patients (493 530 patients without and 10 835 patients with SCA), Black race remained an independent risk factor associated with SCA (OR, 1.10; 95% CI, 1.03-1.17; *P* = .004), whereas Hispanic race was found to be a protective factor associated with decreased odds of SCA (OR, 0.71; 95% CI, 0.67-0.76; *P* < .001). Compared with White patients in the SCA subgroup, Black patients had a 26.5% (95% CI, 11.0%-44.3%) longer length of stay (*P* < .001) for mild to moderate injuries and a 40.1% (95% CI, 16.4%-68.5%) longer length of stay (*P* < .001) for serious injury.

**Conclusion and Relevance:**

In this study, Black children and adolescents were suspected to have experienced child abuse at higher rates than children and adolescents of other racial and ethnic backgrounds. More research is necessary to understand the origins of these disparities to reduce them in child abuse identification.

## Key Points

**Question:**

Are there racial and ethnic disproportionalities in suspicion for child abuse in pediatric patients admitted after traumatic injury?

**Findings:**

In this cross-sectional study using data for 634 309 pediatric patients (weighted observations with complete data) abstracted from the Kids’ Inpatient Database, Black race was an independent risk factor associated with suspicion for child abuse and Hispanic race was a protective factor associated with lower odds of suspicion for child abuse after controlling for socioeconomic factors and hospital characteristics.

**Meaning:**

This study found that Black children and adolescents were suspected to have experienced child abuse at higher rates than children and adolescents of other racial and ethnic backgrounds.

## Teaser

This cross-sectional study investigates disproportionalities by race and ethnicity in suspicion for child abuse among pediatric patients admitted after traumatic injury.

## Introduction

Child abuse, encompassing physical, sexual, and psychological abuse, is a leading cause of early childhood injury and death. In particular, child physical abuse affects 9 of 1000 children and adolescents in the US each year, with an estimated 1750 deaths annually.^1,2^ Risk factors associated with physical child abuse, in particular, have been extensively investigated. Children younger than 1 year, children and adolescents with special needs and chronic illnesses, and children and adolescents from low socioeconomic status (SES) backgrounds have been shown to be at increased risk for physical child abuse and maltreatment.^3,4,5,6,7^ Studies examining poverty in the general child abuse literature have demonstrated that economically insecure children and adolescents experience 3 to 9 times more maltreatment than their economically secure counterparts.^7,8^ Furthermore, male sex has been established as a risk factor associated with childhood maltreatment, especially in cases of physical child abuse.^9,10^ However, race and ethnicity as a risk factor associated with child abuse overall remains controversial.^1,10,11,12,13,14^ Children and adolescents from minority race and ethnicity populations tend to be overrepresented in the child welfare system.^15,16^ As a result, one question that has been raised is whether mandated reporters are more likely to suspect physical child abuse among Black children and adolescents and those with low SES compared with White children and adolescents and those with high SES.^3,17^ The racial and ethnic disparity in physical suspicion for child abuse (SCA) has been shown to be significant even after adjusting for the likelihood of abuse.^17^

Notably, 3 independent analyses of the National Incidence Study^15,18,19^ found no difference in child and adolescent maltreatment with respect to race and ethnicity. Other studies have suggested that cultural differences may affect a clinician’s threshold for reporting any type of child abuse; for example, clinicians may be less likely to report parents with similar ethnicity and SES to the clinician.^15,20^ These findings raise concern that, generally, different thresholds exist for suspicion of child abuse, which may result in the overevaluation of some children and adolescents and underevaluation of others. Overevaluation of Black children and adolescents and those with low SES not only risks unnecessary and traumatic family separation, but may also increase hospital costs, hospitalization length, and caregiver anxiety.^12^ Conversely, underevaluation of White children and adolescents and those with high SES may lead to recurring and escalating injuries from abuse.^3^

Multiple previous studies have used the Kids’ Inpatient Database (KID) to study child physical abuse in particular but have yet to rigorously interrogate the role of race and ethnicity in this population, to our knowledge. Peace et al^21^ used KID to compare children and adolescents with nonaccidental trauma (NAT) with those with accidental trauma, finding that children and adolescents with NAT were less likely to identify as White and more likely to use public insurance. However, the authors did not study race or ethnicity in NAT on multivariable regression analyses. Additional studies have evaluated abusive head trauma, in particular, with KID data but have reported conflicting results with respect to race and ethnicity.^22,23,24^ In this study, we sought to evaluate whether differences existed with respect to race and ethnicity and reporting trends among children and adolescents suspected of experiencing child abuse (encompassing physical, sexual, and psychological child abuse).

## Methods

This cross-sectional study was reviewed by the institutional review board at Stanford University and deemed exempt from review and informed consent because data were deidentified. This study is reported following the Strengthening the Reporting of Observational Studies in Epidemiology (STROBE) reporting guideline.

### Data Source

As part of the Healthcare Cost and Utilization Project (HCUP), the Agency for Healthcare Research and Quality developed KID, the largest public, all-payer database for pediatric inpatient care in the US.^25^ This study examined inpatient admissions for pediatric trauma using KID. The database enables the comparison of hospitalization characteristics among patients with suspected child abuse because it includes data on a representative sample of pediatric discharges from all community, nonrehabilitation hospitals in the US participating in HCUP, encompassing approximately 3 million hospitalizations each year.^4,23,26,27^ KID reports are published every 3 years and summarize data from 48 states and the District of Columbia. Using this database, we assessed patient demographic data and hospitalization characteristics for our population of interest.

Race and ethnicity are included in this study according to the data reported in KID, and importantly, the reporting of information on race and ethnicity can vary by hospital. Additionally, race and ethnicity information are reported in KID as 1 combined field instead of separate fields, as recommended by the National Institutes of Health. Race and ethnicity categories reported included Asian, Black non-Hispanic, Hispanic, Native American (hereafter, *American Indian*), Pacific Islander, and White non-Hispanic. Other refers to a race or ethnicity that is not American Indian, Asian, Black non-Hispanic, Hispanic, Pacific Islander, White non-Hispanic, unknown or not provided, or multiple races or ethnicities (ie, multiple categories selected).

### Study Population

Admissions for traumatic pediatric injury were identified from the 2006, 2009, 2012, 2016, and 2019 KID reports, each including data from 48 states, using *International Classification of Diseases, Ninth Revision, Clinical Modification *(*ICD-9-CM*) and *International Statistical Classification of Diseases, Tenth Revision, Clinical Modification *(*ICD-10-CM*) codes. The following codes were used to identify cases of traumatic injury: *ICD-9-CM* codes 800.00 to 959.0, excluding 905 to 924 and 930 to 949, were used for 2006, 2009, and 2012 reports, and *ICD-10-CM* codes S00 to S99, T07, T14, T20 to T28, and T30 to T32 were used for 2016 and 2019 reports. The SCA subgroup was identified using the following codes: *ICD-9-CM* codes 995.50, 995.51, 995.52, 995.53, 995.54, 995.55, 995.59, and E967.0-967.9 and *ICD-10-CM* codes T74.02XA, T74.1, T74.12XA, T74.22XA T74.32XA, T74.4XXA, T74.9, T74.92XA, T76.02XA, T76.1, T76.12XA, T76.22XA, T76.9A, T76.92XA, V71.81, Y07-Y07.59, and Y07.9. Relevant child abuse codes for this study were identified based on literature review and in discussion with child abuse specialists at our institution.^28,29,30^ We included child abuse codes for physical, sexual, and psychological child abuse, as well as neglect; in combination with concurrent trauma injury codes, this allowed us to capture all potential cases of child and adolescent maltreatment with a special emphasis on physical child abuse. Codes used in this study for identifying the SCA subgroup are further detailed in eTables 1 to 2 in Supplement 1. Of note, we did not include assault codes in our dataset given that it is difficult to delineate differences between adult and peer assault based on age alone.

### Variables

The primary aim of our study was to investigate whether racial and ethnic disproportionalities existed in the reporting of SCA. To do so, we compared the racial and ethnic composition of patients who had SCA trauma with that of patients who had non-SCA trauma, as well as the US population overall using data provided by the 2010 US Census.^31^ The secondary aim of our study was to compare whether differences existed based on population demographics, injury severity score (ISS), or hospitalization characteristics between SCA and non-SCA subgroups. Demographic data queried from the KID database included race and ethnicity, sex, age (stratified into 5 groups: ages <1, 1-3, 4-7, 8-11, and 12-17 years), insurance information (characterized as Medicaid, private, self-pay, or other), median household income by zip code as a proxy for SES (divided into 4 quartiles: $ 1-$40 999, $41 000-$50 999, $51 000-$66 999, and ≥$67 000), and ISS (categorized as mild to moderate [1-15], serious [16-24], and severe [≥25]). Admission characteristics of interest to our study included hospital bed size (small, medium, or large), hospital region (Northeast, Midwest, South, or West), hospital setting (rural, urban nonteaching, or urban teaching), admission length of stay (LOS), mortality, and hospitalization cost. Hospital size definition based on region is presented in eTable 3 in Supplement 1.

### Statistical Analysis

Categorical variables are presented as frequencies and percentages and compared using the Fisher exact test. Continuous variables are represented as median and IQR, with comparisons performed using the Mann-Whitney *U* test, a nonparametric hypothesis test. Statistical significance was defined as a 2-sided *P* < .05. Statistical analysis was performed between March 2022 and October 2024 using R statistical software version 4.1.2 (R Project for Statistical Computing). We excluded records with missing data on regression model inputs (sex, age, ISS, payer, median household income by zip code, hospital factors [teaching status, bed size, or region], mortality, and hospital LOS) (eFigure in Supplement 1). The multivariable regression model included only patients in the 3 largest race and ethnicity groups: Black, White, and Hispanic.

Univariate regression analysis was performed to compare the racial and ethnic composition of the SCA population with that of the non-SCA population (Black, Hispanic, or White). Next, we performed multivariate logistic regression analyses in SCA and non-SCA subgroups to evaluate the association of race and ethnicity as a factor after controlling for other confounding factors (eg, demographic characteristics, ISS, and hospitalization status). We then performed an additional multivariate analysis assessing the association of race and ethnicity with mortality rates and LOS comparing SCA and non-SCA subgroups. Notably, due to the skewed distribution of LOS, we used log linear models with γ family and log link to estimate factors. California Code of Regulations codes and total charges were used to calculate the total cost of hospitalization. To normalize across years, total cost was adjusted to 2021 prices using the Consumer Price Index. Importantly, to account for KID’s complex sampling scheme, all analyses are performed using weighted data..

## Results

### Patient Demographic Characteristics

An unweighted total of 466 781 pediatric patients admitted after traumatic injury younger than 18 years were identified from 2006, 2009, 2012, 2016, and 2019 KID registries. The total weighted population resulted in 675 007 patients (eFigure in Supplement 1). There were 342 753 pediatric patients in the non-SCA trauma subgroup and 7494 pediatric patients in the SCA trauma subgroup prior to weighting. Among 634 309 pediatric patients after weighting with complete data, 13 579 patients had injuries attributable to SCA (SCA subgroup; mean [SD] age, 1.70 [0.04] years; 7650 male [56.3%]; 133 American Indian [1.0%], 133 Asian or Pacific Islander [1.0%], 2868 Black [21.1%], 2293, Hispanic [16.9%], 5675 White [41.8%], and 687 other race or ethnicity [5.1%]) and 620 730 patients did not (non-SCA subgroup; mean [SD] age, 9.70 [0.01] years; 395 158 male [63.7%]; 5128 American Indian [0.8%], 13 479 Asian or Pacific Islander [2.2%], 86 376 Black [13.9%], 108 406 Hispanic [17.5%], 298 748 White [48.1%], and 27 680 other race or ethnicity [4.5%]) (Table 1). For our downstream regression analyses including only Black, Hispanic, and White patients, a weighted total of 504 365 patients, including 493 530 patients without SCA and 10 835 patients with SCA, were analyzed. Accordingly, the SCA subgroup accounted for 2.1% of pediatric patients admitted after traumatic injury after weighting.

**Table 1.** Baseline Demographics and Hospitalization Characteristics

Characteristic	Patients, No. (%) (N = 634 309)^a^		*P* value
	Non-SCA (n = 620 730)	SCA (n = 13 579)	
Age, y			
<1	53 486 (8.6)	8448 (62.2)	<.001
1-3	83 392 (13.4)	3413 (25.1)	
4-7	98 556 (15.9)	665 (4.9)	
8-11	79 497 (12.8)	252 (1.9)	
12-17	305 799 (49.3)	800 (5.9)	
Median (IQR)	11.0 (4.0 to 15.0)	<0.1 (<0.1 to 2.0)	<.001
Mean (SD)	9.70 (0.01)	1.70 (0.04)	<.001
Sex			
Male	395 158 (63.7)	7650 (56.3)	<.001
Female	225 572 (36.3)	5929 (43.7)	
Race and ethnicity			
American Indian	5128 (0.8)	133 (1.0)	<.001
Asian or Pacific Islander	13 479 (2.2)	133 (1.0)	
Black	86 376 (13.9)	2868 (21.1)	
Hispanic	108 406 (17.5)	2293 (16.9)	
White	298 748 (48.1)	5675 (41.8)	
Other^b^	27 680 (4.5)	687 (5.1)	
Not recorded	80 913 (13.0)	1791 (13.2)	
ISS			
0-15	510 601 (82.3)	7311 (53.8)	<.001
16-24	60 021 (9.7)	3225 (23.7)	
>25	50 108 (8.1)	3043 (22.4)	
Median (IQR)	4.0 (2.0 to 10.0)	13.0 (5.0 to 22.0)	<.001
Median household income quartile			
First ($1-$40 999)	189 008 (30.4)	5221 (38.5)	<.001
Second ($41 000-$50 999)	152 689 (24.6)	3730 (27.5)	
Third ($51 000-$66 999)	146 128 (23.5)	3046 (22.4)	
Fourth (≥$67 000)	132 905 (21.4)	1581 (11.6)	
Primary payer			
Medicaid	260 425 (42.0)	10 406 (76.6)	<.001
Private	300 148 (48.4)	2162 (15.9)	
Self-pay	29 287 (4.7)	369 (2.7)	
Other	30 870 (5.0)	642 (4.7)	
Hospital size^c^			
Small	64 576 (10.4)	1794 (13.2)	<.001
Medium	134 220 (21.6)	2815 (20.7)	
Large	421 934 (68.0)	8970 (66.1)	
Hospital type			
Rural	30 300 (4.9)	250 (1.8)	<.001
Urban nonteaching	94 274 (15.2)	743 (5.5)	
Urban teaching	496 156 (79.9)	12 586 (92.7)	
Hospital region			
Northeast	109 996 (17.7)	2019 (14.9)	<.001
Midwest	135 360 (21.8)	3617 (26.6)	
South	223 189 (36)	5118 (37.7)	
West	152 185 (24.5)	2825 (20.8)	
Mortality	6496 (1.0)	761 (5.6)	<.001
LOS, median (IQR), d	2.0 (1.0 to 4.0)	3.0 (2.0 to 8.0)	<.001
Total cost, median (IQR), $	7306.3 (4050.6 to 13 930.0)	9365.8 (4546.7 to 23 345.8)	<.001
Not recorded	15 351 (2.5)	373 (2.7)	
Daily cost, median (IQR), $	3504.4 (2059.7 to 5668.4)	2696.5 (1740.6 to 4199.2)	<.001
Not recorded	15 401 (2.5)	373.9 (2.7)	

Abbreviations: ISS, injury severity score; LOS, length of stay; SCA, suspected child abuse.

^a^ Some rows may sum to more or less than 634 309 due to rounding errors from weighting.

^b^ Other refers to a race or ethnicity that is not American Indian, Asian, Black non-Hispanic, Hispanic, Pacific Islander, White non-Hispanic, unknown or not provided, or multiple races or ethnicities (ie, multiple categories selected).

^c^ Hospital size definition based on the region is presented in eTable 3 in Supplement 1.

### SCA vs Non-SCA Subgroup

Baseline characteristics for SCA and non-SCA subgroups are provided in Table 1. Compared with the non-SCA subgroup, the SCA subgroup was younger. Differences observed in racial and ethnic distribution between non-SCA and SCA subgroups were significant. As a comparison, the distribution of race and ethnicity in the US population based on the 2010 Census was 1% American Indian, 5% Asian or Pacific Islander, 13% Black, 16% Hispanic, 56% White, and 9% other.^31^ Based on these data, the distribution of race and ethnicity with respect to Black patients in the non-SCA subgroup was relatively concordant with the distribution reported by the 2010 US Census. However, in the SCA subgroup, White patients were relatively underrepresented and Black and Hispanic patients were overrepresented compared with the US Census. Hispanic patients were overrepresented in the non-SCA subgroup as well.

Next, we evaluated the association of SES with SCA in the weighted population with complete data. A higher proportion of patients in the SCA compared with non-SCA subgroup came from families with low SES as defined by the lowest median household income quartile (5221 patients [38.5%] vs 189 008 patients [30.4%]; *P* < .001), and a lower proportion came from families with high SES as defined by the highest median household income quartile (1581 patients [11.6%] vs 132 905 patients [21.4%]; *P* < .001). Most of the SCA subgroup was insured by Medicaid (10 406 patients [76.6%]), with the remaining portion predominantly insured by private insurance (2162 patients [15.9%]). However, most patients in the non-SCA subgroup had private insurance (300 148 patients [48.4%]), with a smaller proportion insured by Medicaid (260 425 patients [42.0%]) (*P* < .001). Although both groups presented to teaching hospitals most frequently, a greater percentage of the SCA subgroup was seen in this setting compared with the non-SCA subgroup (12 586 patients [92.7%] vs 496 156 patients [79.9%]; *P* < .001).

Additionally, we investigated the association of ISS with SCA. The SCA subgroup tended to present with more severe injuries and was noted to have higher mortality rates and LOS. The SCA subgroup had a median (IQR) ISS of 13.0 (5.0-22.0) compared with 4.0 (2.0-10.0) in the non-SCA subgroup (*P* < .001). Additionally, the SCA subgroup had a higher mortality rate (761 patients [5.6%] vs 6496 patients [1.0%]; *P* < .001) and a longer median (IQR) LOS (3.0 [2.0-8.0] days vs 2.0 [1.0-4.0] days; *P* < .001). Although the median (IQR) total hospitalization cost for the SCA subgroup was higher than that of the non-SCA subgroup ($9375.1 [$4546.4-$23 354.2] vs $7305.0 [$4050.4-$13 923.8]), the median (IQR) daily cost for the SCA subgroup was lower than that of the non-SCA subgroup ($2696.8 [$1739.9-$4200.0] vs $3504.5 [$2060.1-$5668.6]; *P* < .001).

### Characterization and Analysis of SCA Subgroup

Next, we stratified the SCA subgroup by race and ethnicity to perform downstream analyses, focusing on Black (2868 patients), Hispanic (2293 patients), and White (5675 patients) subpopulations (Table 2). These analyses showed that there were more males than females in all race and ethnicity groups analyzed (3231 Black males [56.9%]; 1647 Hispanic males [57.4%]; 1278 White males [55.7%]) (Table 2). The HCUP database prohibits researchers from publishing data on subgroups with cell size less than or equal to 10; as such, we omitted all other racial subgroups from our regression analyses to comply with HCUP policy. Regression analyses were then performed to interrogate the association between race and ethnicity and SCA (Table 3). Univariate logistic regression of race and ethnicity as a factor associated with SCA revealed higher odds of SCA for Black (odds ratio [OR], 1.75; 95% CI, 1.65-1.85; *P* < .001) and Hispanic (OR, 1.11; 95% CI, 1.05-1.18; *P* < .001) compared with White patients admitted after traumatic injury. After controlling for other factors (eg, age, sex, SES, insurance payer, ISS, and hospitalization characteristics) via multivariate logistic regression analysis, Black racial identity remained an independent risk factor associated with SCA, although with a relatively decreased OR after multivariate analysis (OR, 1.10; 95% CI, 1.03-1.17; *P* = .004). It is possible that there were other potentially confounding variables available in our dataset, and if so, the OR for Black racial identity could decrease further. In contrast, Hispanic racial identity was associated with decreased odds for SCA after controlling for other factors (OR, 0.71; 95% CI, 0.67-0.76; *P* < .001) (Table 3).

**Table 2.** Stratification of SCA Subgroup by Race and Ethnicity

Characteristic	Patients with SCA, No. (%) (N = 10 835)^a^			*P* value
	Black (n = 2868)	Hispanic (n = 2293)	White (n = 5675)	
Age, y				
<1	3580 (63.1)	1637 (57.1)	1424 (62.1)	<.001
1-3	1409 (24.8)	809 (28.2)	573 (25.0)	
4-7	311 (5.5)	182 (6.3)	162 (7.1)	
8-11	265 (4.7)	181 (6.3)	93 (4.0)	
12-17	110 (1.9)	59 (2.0)	40 (1.7)	
Median (IQR)	0 (0-2)	0 (0-2)	0 (0-3)	
Sex				
Male	3231 (56.9)	1647 (57.4)	1278 (55.7)	.67
Female	2444 (43.1)	1221 (42.6)	1015 (44.3)	
ISS				
0-15	3056 (53.8)	1657 (57.8)	1236 (53.9)	<.001
16-24	1385 (24.4)	560 (19.5)	544 (23.7)	
>25	1234 (21.7)	651 (22.7)	513 (22.4)	
Median (IQR)	12 (4-22)	13 (5-22)	12 (4-17)	
Median household income quartile				
First ($1-$40 999)	1808 (31.9)	1527 (53.2)	956 (41.7)	<.001
Second ($41 000-$50 999)	1752 (30.9)	644 (22.4)	565 (24.6)	
Third ($51 000-$66 999)	1387 (24.4)	475 (16.6)	530 (23.1)	
Fourth (≥$67 000)	728 (12.8)	222 (7.7)	243 (10.6)	
Primary payer				
Medicaid	4134 (72.8)	2356 (82.2)	1821 (79.4)	<.001
Private	1109 (19.5)	312 (10.9)	270 (11.8)	
Self-pay	129 (2.3)	89 (3.1)	69 (3.0)	
Other	304 (5.4)	111 (3.9)	132 (5.8)	
Hospital size^b^				
Small	684 (12.1)	455 (15.9)	318 (13.9)	<.001
Medium	1184 (20.9)	615 (21.4)	468 (20.4)	
Large	3806 (67.1)	1798 (62.7)	1507 (65.7)	
Hospital type				
Rural	168 (3.0)	15 (0.5)	12 (0.5)	<.001
Urban nonteaching	306 (5.4)	128 (4.5)	164 (7.1)	
Urban teaching	5201 (91.6)	2725 (95.0)	2117 (92.3)	
Hospital region				
Northeast	890 (15.7)	514 (17.9)	360 (15.7)	<.001
Midwest	1697 (29.9)	721 (25.2)	252 (11.0)	
South	2047 (36.1)	1378 (48.1)	744 (32.4)	
West	1041 (18.3)	254 (8.8)	938 (40.9)	
Mortality	281 (5.0)	154 (5.4)	112 (4.9)	.65
LOS, median (IQR), d	3.0 (2.0-7.0)	4.0 (2.0-8.0)	3.0 (2.0-8.0)	<.001
Total cost, median (IQR), $	8454.5 (4078.1-19 963.2)	9580.8 (4732.1-23 886.7)	10 552.6 (5075.4-26 038.1)	<.001
Not recorded	157 (2.8)	130 (4.5)	42 (1.8)	
Daily cost, median (IQR), $	2657.1 (1711.4-4112.4)	2534.5 (1663.4-3967.9)	2971.0 (1934.2-4582.7)	<.001
Not recorded	157 (2.8)	130 (4.5)	42 (1.8)	

Abbreviations: ISS, injury severity score; LOS, length of stay; SCA, suspected child abuse.

^a^ Some rows may sum to more or less than 10 835 due to rounding error from weighting.

^c^ Hospital size definition based on the region is presented in eTable 3 in Supplement 1.

**Table 3.** Association of Various Factors With SCA

Variables	SCA, OR (95% CI)	*P* value
**Univariate logistic regression of race and ethnicity**		
Black vs White	1.75 (1.65-1.85)	<.001
Hispanic vs White	1.11 (1.05-1.18)	<.001
**Multivariate logistic regression analysis**		
Race and ethnicity		
Black vs White	1.10 (1.03-1.17)	.004
Hispanic vs White	0.71 (0.67-0.76)	<.001
Age, y		
<1 vs 12-17	41.31 (37.60-45.39)	<.001
1-3 vs 12-17	12.28 (11.12-13.57)	<.001
4-7 vs 12-17	2.38 (2.09-2.72)	<.001
8-11 vs 12-17	1.16 (0.97-1.39)	.10
Sex: female vs male	1.11 (1.06-1.17)	<.001
ISS		
0-15 vs >25	0.37 (0.34-0.39)	<.001
16-24 vs >25	1.02 (0.95-1.11)	.52
Median household income quartile		
First vs fourth	1.23 (1.12-1.34)	<.001
Second vs fourth	1.26 (1.15-1.37)	<.001
Third vs fourth	1.26 (1.15-1.37)	<.001
Primary payer		
Private vs Medicaid	0.33 (0.31-0.35)	<.001
Self-pay vs Medicaid	0.56 (0.48-0.65)	<.001
Other vs Medicaid	0.84 (0.75-0.94)	.003
Hospital size^a^		
Medium vs small	0.88 (0.80-0.95)	.003
Large vs small	0.83 (0.77-0.89)	<.001
Hospital type		
Urban nonteaching vs rural	0.96 (0.77-1.18)	.68
Urban teaching vs rural	1.89 (1.56-2.29)	<.001
Hospital region		
Midwest vs Northwest	1.21 (1.12-1.31)	<.001
South vs Northwest	0.91 (0.85-0.98)	.02
West vs Northwest	0.98 (0.91-1.07)	.71

Abbreviations: ISS, injury severity score; OR, odds ratio; SCA, suspected child abuse.

^a^ Hospital size definition based on the region is presented in eTable 3 in Supplement 1.

While there was a greater proportion of male patients in the SCA subgroup across all 3 racial and ethnic groups analyzed, female sex was associated with a higher odds of SCA vs male sex (OR, 1.11; 95% CI, 1.06-1.17; *P* < .001) after controlling for all other variables. Additionally, children younger than 1 year (OR, 41.31; 95% CI, 37.60-45.39; *P* < .001) or aged 1 to 3 years (OR, 12.28; 95% CI, 11.12-13.57; *P* < .001) had higher odds of SCA compared with patients aged 12 to 17 years. Lowest quartile SES was also associated with increased odds of SCA (OR, 1.23; 95% CI, 1.12-1.34; *P* < .001) compared with patients from the highest SES quartile. Private (OR, 0.33; 95% CI, 0.31-0.35; *P* < .001) and self-pay (OR, 0.56; 95% CI, 0.48-0.65; *P* < .001) insurance were associated with lower odds of SCA compared with Medicaid.

### Multivariate Analysis of In-Hospital Mortality and LOS

Next, we performed multivariate regression analyses to examine in-hospital mortality adjusted for race and ethnicity within the overall study group and SCA and non-SCA subgroups (Table 4). The overall OR for mortality in Black vs White patients admitted after traumatic injury was 1.18 (95% CI. 1.08-1.28; *P* < .001), and the OR for mortality in Hispanic vs White patients admitted after traumatic injury was 0.94 (95% CI, 0.86-1.03; *P* = .23), which was not statistically significant. Multivariate regression analysis of mortality in the non-SCA subgroup demonstrated a racial disparity in the OR for mortality, with Black racial identity being associated with higher odds of mortality compared with White racial identity (OR, 1.18; 95% CI, 1.08-1.29; *P* < .001). In the SCA subgroup, there was no significant difference in the odds for mortality between Black and White identity or between Hispanic and White identity (Table 4).

**Table 4.** Risk of In-Hospital Mortality by Race and Ethnicity

Race and ethnicity	Overall population		Non-SCA		SCA	
	OR (95% CI)	*P* value	OR (95% CI)	*P* value	OR (95% CI)	*P* value
Black vs White	1.18 (1.08-1.28)	<.001	1.18 (1.08-1.29)	<.001	1.02 (0.78-1.34)	.89
Hispanic vs White	0.94 (0.86-1.03)	.23	0.96 (0.87-1.06)	.45	0.97 (0.71-1.32)	.84

Abbreviations: OR, odds ratio; SCA, suspected child abuse.

Additionally, we analyzed the association of race and ISS with LOS in SCA and non-SCA subgroups (Table 5). Black patients with mild to moderate ISS and serious ISS demonstrated 26.5% (95% CI, 11.0%-44.3%; *P* *<* .001) and 40.1% (95% CI, 16.4%-68.5%; *P* *<* .001), respectively, longer LOS compared with White patients in the same ISS categories in the SCA subgroup. A similar multivariate regression analysis on the non-SCA subgroup revealed that Black patients with mild to moderate ISS, serious ISS, and severe ISS had 6.3% (95% CI, 3.7%-9.0%), 17.8% (95% CI, 11.2%-24.5%), and 20.0% (95% CI, 14.1%-26.0%), respectively, higher LOS than White patients in the same ISS categories (all *P* *<* .001).

**Table 5.** Increase in LOS for Black vs White Patients by ISS

ISS	Non-SCA		SCA	
	Increase in LOS (95% CI), %	*P* value	Increase in LOS (95% CI), %	*P* value
Mild to moderate (0-15)	6.3 (3.7-9.0)	<.001	26.5 (11.0-44.3)	<.001
Serious (16-24)	17.8 (11.2-24.5)	<.001	40.1 (16.4-68.5)	<.001
Severe (≥25)	20.0 (14.1-26.0)	<.001	17.3 (1.2-36.0)	.054

Abbreviations: ISS, injury severity score; OR, odds ratio; SCA, suspected child abuse.

## Discussion

Using KID-derived data encompassing 2006 to 2019 inpatient pediatric trauma admissions, this cross-sectional study identified Black race as an independent risk factor associated with SCA (encompassing physical, sexual, and psychological abuse) and Hispanic race as a protective factor associated with lower odds of SCA after controlling for multiple socioeconomic and hospital-associated variables. After categorizing our study population into SCA and non-SCA subgroups, we identified that these subgroups differed in demographics, ISS, and hospitalization characteristics. The SCA subgroup was younger, with most patients younger than 1 year, whereas the non-SCA subgroup consisted predominantly of teenaged patients, with relatively higher SES and most commonly insured by private insurance. Additionally, the SCA subgroup tended to present with higher-severity injuries by ISS. Interestingly, patients in the SCA subgroup were 3 times more likely to present with severe injury by ISS compared with the non-SCA subgroup. The SCA subgroup also had a higher mortality rate than the non-SCA subgroup. In addition to identifying Black race as a factor associated with SCA, our findings validate those reported in previous studies, with higher rates of severe injury and mortality in patients suspected of experiencing child abuse.^2,5,12,32^

There was a discrepancy with respect to race and ethnicity compared with the US population in the distribution of patients suspected of experiencing child abuse. Our findings are similar to those reported by Luken et al,^33^ who studied state-level geographic variability of racial and ethnic disparities for physical, sexual, and emotional abuse in children and adolescents, finding that Black individuals were overall the most overrepresented group compared with other racial and ethnic groups. Additionally, the authors reported that Latinx-identified children and adolescents were underrepresented with respect to all maltreatment populations in approximately half of states in their study. Unconscious bias may perpetuate health care inequity in that diagnostic decision-making may be influenced by racial and ethnic stereotypes.^34^ Suspicion for NAT or child abuse is one such diagnosis that may be heavily influenced by unconscious bias.

Notably, despite a similar median ISS between Black and White patients admitted after traumatic injury, we found that Black patients had higher odds of being suspected of experiencing child abuse. Additionally, a greater proportion of Black patients suspected of experiencing abuse presented with mild to moderate ISS than White patients, suggesting a lower threshold for SCA with for Black patients and a higher threshold for White patients. Previous studies have found that in children and adolescents with indeterminate-severity injuries, reports of SCA were filed in a significantly lower proportion of White patients than patients with racial and ethnic minority backgrounds (22.5% vs 52.9%; *P < *.001).^17^ In line with these findings, Cort et al^19^ reported that children and adolescents in minority racial and ethnic groups with fractures due to NAT were reported to child protective services at a significantly higher rate than that of White children with similar injuries.

Our data suggest that health care clinicians may be more suspicious of child abuse in Black patients with low SES than White patients with low SES. It is possible that White patients with high SES may be overlooked for SCA, especially if they present with less severe injuries, hypothetically putting them at a higher risk of recurrent NAT with more severe injuries in the future. Addressing unconscious bias in the diagnosis of NAT requires comprehensive and evidence-based interventions.^34,35,36^ NAT is generally associated with higher mortality and worse outcomes compared with the other types of traumatic injuries and accounts for 46% of all traumatic deaths in the pediatric population; failure to diagnose NAT is associated with a mortality rate of 10% by the time of the following visit.^32,37,38,39^ Adherence to screening protocols will increase the accuracy and detection of NAT cases.^40,41^ Additionally, workforce diversity in the hospital could also ameliorate the association of unconscious bias with NAT detection and diagnosis.^35^ Importantly, we found that the odds of high mortality were similar between White and Black patients, despite increased odds of SCA abuse in our Black patient population.

Interestingly, the SCA subgroup was more likely than the non-SCA subgroup to have received care at teaching hospitals. These data suggest that child abuse may be more readily detected at teaching hospitals compared with other settings. One potential reason for this may be that teaching hospitals are more likely to teach standard NAT screening guidelines to trainees, which may contribute to higher reports of SCA cases. Additional reasons may be the location of teaching hospitals in urban, low-SES regions, as well as the likelihood that patients experiencing child abuse get transferred to teaching hospitals.

### Limitations

Our study has several limitations. First and foremost, we used KID as our primary data source, so our results are inherently limited by factors that affect most administrative databases. These include a lack of clinical variables, a potential for coding errors, and an inability to follow up with patients over time. The KID database has a single data field combining race and ethnicity, which makes racial differences among Hispanic patients impossible to assess and complicates comparison with other studies that capture race and ethnicity separately. Owing to the retrospective nature of our study and the lack of patient-related data, several confounding variables may affect SES, race and ethnicity, and sex distributions of SCA and non-SCA subgroups. For example, SES in our study was based only on median household income, which may not be completely representative of patient SES. Additionally, KID provides data on hospitalized patients only, which may not be representative of the SCA population overall. Furthermore, different subgroup sizes of the SCA and non-SCA group may affect the accuracy of comparing them to one another. Additionally, low representation of some minority race and ethnicity populations (eg, American Indian and Asian patients) in KID causes difficulty in comparisons among these subgroups.

## Conclusions

This cross-sectional study found that younger patients and patients from low SES backgrounds were more likely to be suspected of experiencing child abuse. In our results, Black patients were more likely to be suspected of experiencing child abuse, and White patients were less likely to be suspected of experiencing it, despite similar median ISS and mortality rates. These outcomes suggest that clinicians may have a lower threshold for SCA with Black patients and a higher threshold with White patients. Black patients admitted after traumatic injury tend to have a longer LOS compared with White patients admitted after traumatic injury, but the difference is more prominent among patients who are suspected of experiencing child abuse. Developing a standardized screening tool may help eliminate unconscious bias in diagnosing NAT among different races and ethnicities.

## Notes

Supplement 1. **eTable 1.** *ICD-9-CM* Child Abuse and Maltreatment Codes **eTable 2.** *ICD-10-CM* Child Abuse and Maltreatment Codes **eTable 3.** Hospital Bed Size Categories Defined by KID **eFigure.** Flowchart Defining Study Criteria for Downstream Analyses

Supplement 2.
Data Sharing Statement

## References

[1] Rosenfeld EH, Johnson B, Wesson DE, Shah SR, Vogel AM, Naik-Mathuria B. Understanding non-accidental trauma in the United States: a national trauma databank study. J Pediatr Surg. 2020;55(4):693-697. doi:10.1016/j.jpedsurg.2019.03.024 31103270

[2] Roaten JB, Partrick DA, Nydam TL, . Nonaccidental trauma is a major cause of morbidity and mortality among patients at a regional level 1 pediatric trauma center. J Pediatr Surg. 2006;41(12):2013-2015. doi:10.1016/j.jpedsurg.2006.08.028 17161194

[3] Wood JN, Hall M, Schilling S, Keren R, Mitra N, Rubin DM. Disparities in the evaluation and diagnosis of abuse among infants with traumatic brain injury. Pediatrics. 2010;126(3):408-414. doi:10.1542/peds.2010-0031 20713477

[4] Trokel M, Discala C, Terrin NC, Sege RD. Patient and injury characteristics in abusive abdominal injuries. Pediatr Emerg Care. 2006;22(10):700-704. doi:10.1097/01.pec.0000238734.76413.d0 17047468

[5] Larimer EL, Fallon SC, Westfall J, Frost M, Wesson DE, Naik-Mathuria BJ. The importance of surgeon involvement in the evaluation of non-accidental trauma patients. J Pediatr Surg. 2013;48(6):1357-1362. doi:10.1016/j.jpedsurg.2013.03.035 23845630

[6] Oliver WJ, Kuhns LR, Pomeranz ES. Family structure and child abuse. Clin Pediatr (Phila). 2006;45(2):111-118. doi:10.1177/000992280604500201 16528430

[7] Austin AE, Lesak AM, Shanahan ME. Risk and protective factors for child maltreatment: a review. Curr Epidemiol Rep. 2020;7(4):334-342. doi:10.1007/s40471-020-00252-3 34141519 PMC8205446

[8] Conrad-Hiebner A, Byram E. The temporal impact of economic insecurity on child maltreatment: a systematic review. Trauma Violence Abuse. 2020;21(1):157-178. doi:10.1177/1524838018756122 29400135

[9] Khan I, Dar IA, Bano S, Iqbal N. Gender differences in childhood maltreatment: a comparative study of nightmare sufferers and non-sufferers. J Child Adolesc Trauma. 2021;14(4):483-491. doi:10.1007/s40653-020-00338-6 34790282 PMC8586107

[10] Lane WG, Dubowitz H, Langenberg P, Dischinger P. Epidemiology of abusive abdominal trauma hospitalizations in United States children. Child Abuse Negl. 2012;36(2):142-148. doi:10.1016/j.chiabu.2011.09.010 22398302 PMC3589583

[11] Brown J, Cohen P, Johnson JG, Salzinger S. A longitudinal analysis of risk factors for child maltreatment: findings of a 17-year prospective study of officially recorded and self-reported child abuse and neglect. Child Abuse Negl. 1998;22(11):1065-1078. doi:10.1016/S0145-2134(98)00087-8 9827312

[12] Paul AR, Adamo MA. Non-accidental trauma in pediatric patients: a review of epidemiology, pathophysiology, diagnosis and treatment. Transl Pediatr. 2014;3(3):195-207. doi:10.3978/j.issn.2224-4336.2014.06.01 26835337 PMC4729847

[13] Escobar MA Jr, Wallenstein KG, Christison-Lagay ER, Naiditch JA, Petty JK. Child abuse and the pediatric surgeon: a position statement from the Trauma Committee, the Board of Governors and the membership of the American Pediatric Surgical Association. J Pediatr Surg. 2019;54(7):1277-1285. doi:10.1016/j.jpedsurg.2019.03.009 30948199

[14] Sonderman KA, Wolf LL, Madenci AL, Beres AL. Insurance status and pediatric mortality in nonaccidental trauma. J Surg Res. 2018;231:126-132. doi:10.1016/j.jss.2018.05.033 30278919

[15] Ards S, Chung C, Myers SL Jr. The effects of sample selection bias on racial differences in child abuse reporting. Child Abuse Negl. 1998;22(2):103-115. doi:10.1016/S0145-2134(97)00131-2 9504213

[16] Crichton KG, Cooper JN, Minneci PC, Groner JI, Thackeray JD, Deans KJ. A national survey on the use of screening tools to detect physical child abuse. Pediatr Surg Int. 2016;32(8):815-818. doi:10.1007/s00383-016-3916-z 27385110

[17] Lane WG, Rubin DM, Monteith R, Christian CW. Racial differences in the evaluation of pediatric fractures for physical abuse. JAMA. 2002;288(13):1603-1609. doi:10.1001/jama.288.13.1603 12350191

[18] Maloney T, Jiang N, Putnam-Hornstein E, Dalton E, Vaithianathan R. Black-White differences in child maltreatment reports and foster care placements: a statistical decomposition using linked administrative data. Matern Child Health J. 2017;21(3):414-420. doi:10.1007/s10995-016-2242-3 28124189

[19] Cort NA, Cerulli C, He H. Investigating health disparities and disproportionality in child maltreatment reporting: 2002-2006. J Public Health Manag Pract. 2010;16(4):329-336. doi:10.1097/PHH.0b013e3181c4d933 20520372 PMC2914097

[20] Hampton RL, Newberger EH. Child abuse incidence and reporting by hospitals: significance of severity, class, and race. Am J Public Health. 1985;75(1):56-60. doi:10.2105/AJPH.75.1.56 3966600 PMC1646161

[21] Peace AE, Caruso D, Agala CB, . Cost of pediatric trauma: a comparison of non-accidental and accidental trauma in pediatric patients. J Surg Res. 2023;283:806-816. doi:10.1016/j.jss.2022.08.045 36470207 PMC10276928

[22] Theodorou CM, Nuño M, Yamashiro KJ, Brown EG. Increased mortality in very young children with traumatic brain injury due to abuse: a nationwide analysis of 10,965 patients. J Pediatr Surg. 2021;56(6):1174-1179. doi:10.1016/j.jpedsurg.2021.02.044 33752910 PMC8131228

[23] Nuño M, Ugiliweneza B, Bardini RL, Ozturk A, Stephenson JT, Magaña JN. Age-related mortality in abusive head trauma. J Trauma Acute Care Surg. 2019;87(4):827-835. doi:10.1097/TA.0000000000002255 30865156

[24] Niederkrotenthaler T, Xu L, Parks SE, Sugerman DE. Descriptive factors of abusive head trauma in young children–United States, 2000-2009. Child Abuse Negl. 2013;37(7):446-455. doi:10.1016/j.chiabu.2013.02.002 23535075

[25] Healthcare Cost and Utilization Project. Kids’ Inpatient Database (KID). Accessed November 5, 2024. https://hcup-us.ahrq.gov/db/nation/kid/kiddbdocumentation.jsp

[26] Leventhal JM, Martin KD, Gaither JR. Using US data to estimate the incidence of serious physical abuse in children. Pediatrics. 2012;129(3):458-464. doi:10.1542/peds.2011-1277 22311999

[27] Lane WG, Lotwin I, Dubowitz H, Langenberg P, Dischinger P. Outcomes for children hospitalized with abusive versus noninflicted abdominal trauma. Pediatrics. 2011;127(6):e1400-e1405. doi:10.1542/peds.2010-2096 21555490 PMC3103272

[28] Schnitzer PG, Slusher P, Van Tuinen M. Child maltreatment in Missouri: combining data for public health surveillance. Am J Prev Med. 2004;27(5):379-384. doi:10.1016/j.amepre.2004.08.007 15556737

[29] Deans KJ, Thackeray J, Askegard-Giesmann JR, Earley E, Groner JI, Minneci PC. Mortality increases with recurrent episodes of nonaccidental trauma in children. J Trauma Acute Care Surg. 2013;75(1):161-165. doi:10.1097/TA.0b013e3182984831 23940863

[30] Schnitzer PG, Slusher PL, Kruse RL, Tarleton MM. Identification of *ICD* codes suggestive of child maltreatment. Child Abuse Negl. 2011;35(1):3-17. doi:10.1016/j.chiabu.2010.06.008 21316104

[31] Fang Y, Jawitz JW. High-resolution reconstruction of the United States human population distribution, 1790 to 2010. Sci Data. 2018;5:180067. doi:10.1038/sdata.2018.67 29688219 PMC5914287

[32] Davies FC, Coats TJ, Fisher R, Lawrence T, Lecky FE. A profile of suspected child abuse as a subgroup of major trauma patients. Emerg Med J. 2015;32(12):921-925. doi:10.1136/emermed-2015-205285 26598630 PMC4717353

[33] Luken A, Nair R, Fix RL. On racial disparities in child abuse reports: exploratory mapping the 2018 NCANDS. Child Maltreat. 2021;26(3):267-281. doi:10.1177/10775595211001926 33729016 PMC8328863

[34] Marcelin JR, Siraj DS, Victor R, Kotadia S, Maldonado YA. The impact of unconscious bias in healthcare: how to recognize and mitigate it. J Infect Dis. 2019;220(220)(suppl 2):S62-S73. doi:10.1093/infdis/jiz214 31430386

[35] Johnson TJ, Ellison AM, Dalembert G, . Implicit bias in pediatric academic medicine. J Natl Med Assoc. 2017;109(3):156-163. doi:10.1016/j.jnma.2017.03.003 28987244 PMC5710818

[36] FitzGerald C, Hurst S. Implicit bias in healthcare professionals: a systematic review. BMC Med Ethics. 2017;18(1):19. doi:10.1186/s12910-017-0179-8 28249596 PMC5333436

[37] Yu YR, DeMello AS, Greeley CS, Cox CS, Naik-Mathuria BJ, Wesson DE. Injury patterns of child abuse: experience of two level 1 pediatric trauma centers. J Pediatr Surg. 2018;53(5):1028-1032. doi:10.1016/j.jpedsurg.2018.02.043 29523358

[38] Chang DC, Knight VM, Ziegfeld S, Haider A, Paidas C. The multi-institutional validation of the new screening index for physical child abuse. J Pediatr Surg. 2005;40(1):114-119. doi:10.1016/j.jpedsurg.2004.09.019 15868569

[39] Quiroz HJ, Parreco J, Easwaran L, . Identifying populations at risk for child abuse: a nationwide analysis. J Pediatr Surg. 2020;55(1):135-139. doi:10.1016/j.jpedsurg.2019.09.069 31757508 PMC7848807

[40] Louwers EC, Korfage IJ, Affourtit MJ, . Accuracy of a screening instrument to identify potential child abuse in emergency departments. Child Abuse Negl. 2014;38(7):1275-1281. doi:10.1016/j.chiabu.2013.11.005 24325939

[41] Louwers EC, Korfage IJ, Affourtit MJ, . Effects of systematic screening and detection of child abuse in emergency departments. Pediatrics. 2012;130(3):457-464. doi:10.1542/peds.2011-3527 22926179

