# Supplementary material for: Racial and Ethnic Disparities in Child Abuse Identification and Inpatient Treatment
Source: JAMA Netw Open. 2024 Dec 18;7(12):e2451588. doi: 10.1001/jamanetworkopen.2024.51588 (PMC11656268; doi:10.1001/jamanetworkopen.2024.51588)

Supplemental Online Content

Salimi-Jazi F, Liang NE, Huang Z, et al. Racial and ethnic disparities in child abuse identification and inpatient treatment. *JAMA Netw Open*. 2024;7(12):e2451588. doi:10.1001/jamanetworkopen.2024.51588

eTable 1. *ICD-9-CM* Child Abuse and Maltreatment Codes
eTable 2. *ICD-10-CM* Child Abuse and Maltreatment Codes
eTable 3. Hospital Bed Size Categories Defined by KID
eFigure. Flowchart Defining Study Criteria for Downstream Analyses

This supplemental material has been provided by the authors to give readers additional information about their work.

© 2024 Salimi-Jazi F et al. *JAMA Netw Open*.

eTable 1. ICD-9-CM Child Abuse and Maltreatment Codes

995.50: child abuse, unspecified
995:51: child emotional/psychological abuse
955.52: child neglect: nutritional
995:53: child sexual abuse
995.54: child physical abuse
995.55: shaken baby syndrome
995.59: other child abuse and neglect
E967.0: perpetrator of child/adult abuse, by father, stepfather or boyfriend
E967.1: perpetrator of child/adult abuse, by other unspecified person
E967.2: perpetrator of child/adult abuse, by mother, stepmother, or girlfriend
E967.3: perpetrator of child/adult abuse, by spouse or partner
E967.4: perpetrator of child/adult abuse, by child
E967.5: perpetrator of child/adult abuse, by sibling
E967.6: perpetrator of child/adult abuse, by grandparent
E967.7: perpetrator of child/adult abuse, by other relative
E967.8: perpetrator of child/adult abuse, by nonrelated caregiver
E967.9: perpetrator of child/adult abuse, by unspecified person

ICD, International Classification of Disease.

© 2024 Salimi-Jazi F et al. *JAMA Netw Open*.

eTable 2. ICD-10-CM Child Abuse and Maltreatment Codes

T74.02X: adult and child abuse, neglect and other maltreatment, confirmed
T74.1: adult and child abuse, neglect and other maltreatment, physical abuse, confirmed
T74.12XA: child physical abuse, confirmed, initial encounter
T74.22XA: child sexual abuse, confirmed, initial encounter
T74.32XA: child psychological abuse, confirmed, initial encounter
T74.4XXA: shaken infant syndrome, initial encounter
T74.9: adult and child abuse, unspecified maltreatment, confirmed
T74.92XA: unspecified child maltreatment, confirmed, initial encounter
T76.02XA: child neglect or abandonment, suspected, initial encounter
T76.1: child physical abuse, suspected
T76.12XA: child physical abuse, suspected, initial encounter
T76.22XA: child sexual abuse, suspected, initial encounter
T76.9A: adult and child abuse, neglect and other maltreatment, suspected
T76.92XA: unspecified child maltreatment, suspected, initial encounter
V71.81: observation and evaluation for abuse and neglect
Y07-Y07.59: perpetrator of assault, maltreatment or neglect
Y-07.9: unspecified perpetrator of maltreatment or neglect

ICD, International Classification of Disease.

© 2024 Salimi-Jazi F et al. *JAMA Netw Open*.

eTable 3. Hospital Bedsize Categories defined by KID.

BEDSIZE CATEGORIES (Beginning in 2000)			
Location and Teaching Status	Hospital Bedsize		
	Small	Medium	Large
**NORTHEAST REGION**			
Rural	1-49	50-99	100+
Urban, nonteaching	1-124	125-199	200+
Urban, teaching	1-249	250-424	425+
**MIDWEST REGION**			
Rural	1-29	30-49	50+
Urban, nonteaching	1-74	75-174	175+
Urban, teaching	1-249	250-374	375+
**SOUTHERN REGION**			
Rural	1-39	40-74	75+
Urban, nonteaching	1-99	100-199	200+
Urban, teaching	1-249	250-449	450+
**WESTERN REGION**			
Rural	1-24	25-44	45+
Urban, nonteaching	1-99	100-174	175+
Urban, teaching	1-199	200-324	325+

© 2024 Salimi-Jazi F et al. *JAMA Netw Open*.

eFigure. Flowchart Defining Study Criteria for Downstream Analyses.

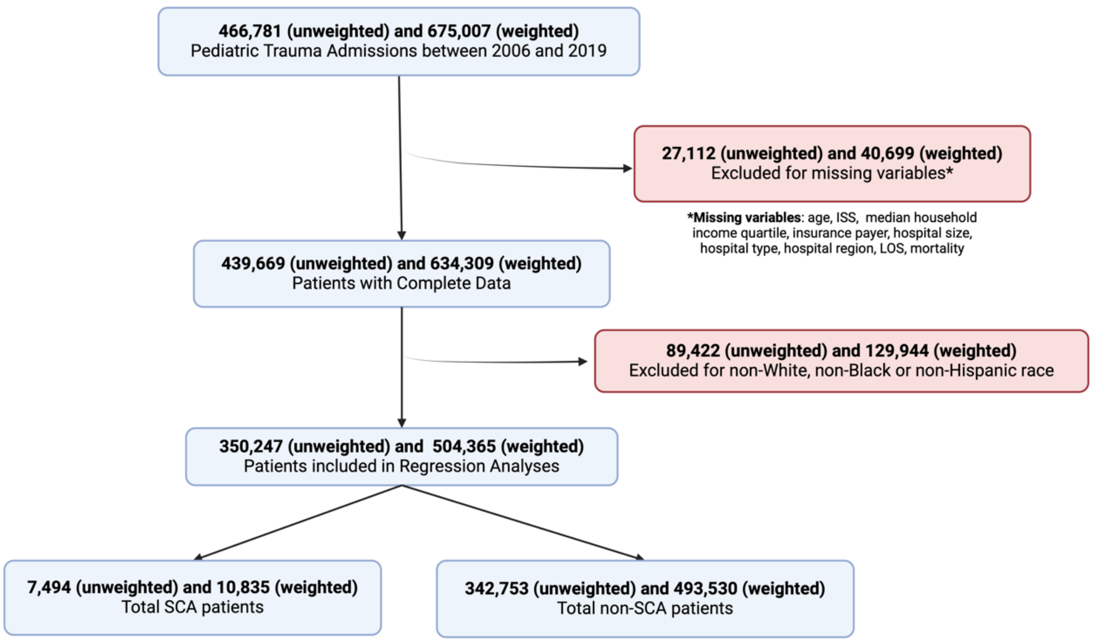

Supplement: Supplement 1. — eTable 1. ICD-9-CM Child Abuse and Maltreatment Codes eTable 2. ICD-10-CM Child Abuse and Maltreatment Codes eTable 3. Hospital Bed Size Categories Defined by KID eFigure. Flowchart Defining Study Criteria for Downstream Analyses [file jamanetwopen-e2451588-s001.pdf]
