# Supplementary material for: Racial and Ethnic Disparities in Child Abuse Identification and Inpatient Treatment
Source: JAMA Netw Open. 2024 Dec 18;7(12):e2451588. doi: 10.1001/jamanetworkopen.2024.51588 (PMC11656268; doi:10.1001/jamanetworkopen.2024.51588)
Supplement: Supplement 2. — Data Sharing Statement [file jamanetwopen-e2451588-s002.pdf]

Data Sharing Statement

Salimi-Jazi. Racial Disparities in Child Abuse Identification and Inpatient Treatment. *JAMA Netw Open*. Published online December 18, 2024. doi:10.1001/jamanetworkopen.2024.51588

Data
Data available: No

Additional Information
Explanation for why data not available: These data are publicly available, with accessed controlled by HCUP.
